# Hyperpolarized ^13^C Magnetic Resonance Imaging of Fumarate Metabolism by Parahydrogen‐induced Polarization: A Proof‐of‐Concept *in vivo* Study

**DOI:** 10.1002/cphc.202001038

**Published:** 2021-03-18

**Authors:** Neil J. Stewart, Hitomi Nakano, Shuto Sugai, Mitsushi Tomohiro, Yuki Kase, Yoshiki Uchio, Toru Yamaguchi, Yujirou Matsuo, Tatsuya Naganuma, Norihiko Takeda, Ikuya Nishimura, Hiroshi Hirata, Takuya Hashimoto, Shingo Matsumoto

**Affiliations:** ^1^ Division of Bioengineering & Bioinformatics Graduate School of Information Science & Technology Hokkaido University North 14, West 9, Kita-ku, Sapporo Hokkaido 060-0814 Japan; ^2^ Division of Computational Chemistry Transition State Technology Co. Ltd. 2-16-1 Tokiwadai, Ube Yamaguchi 755-8611 Japan; ^3^ R&D Department Japan REDOX Ltd. 4-29-49-805 Chiyo, Hakata-ku Fukuoka 812-0044 Japan; ^4^ Division of Cardiology and Metabolism Center for Molecular Medicine Jichi Medical University 3311-1 Yakushiji, Shimotsuke-shi Tochigi 329-0498 Japan; ^5^ Chiba Iodine Resource Innovation Center and Department of Chemistry Graduate School of Science Chiba University 1-33 Yayoi-cho, Inage-ku Chiba 263-8522 Japan

**Keywords:** density functional calculations, fumarate, hyperpolarized ^13^C MRS/I, matabolism, parahydrogen-induced polarization

## Abstract

Hyperpolarized [1‐^13^C]fumarate is a promising magnetic resonance imaging (MRI) biomarker for cellular necrosis, which plays an important role in various disease and cancerous pathological processes. To demonstrate the feasibility of MRI of [1‐^13^C]fumarate metabolism using parahydrogen‐induced polarization (PHIP), a low‐cost alternative to dissolution dynamic nuclear polarization (dDNP), a cost‐effective and high‐yield synthetic pathway of hydrogenation precursor [1‐^13^C]acetylenedicarboxylate (ADC) was developed. The *trans*‐selectivity of the hydrogenation reaction of ADC using a ruthenium‐based catalyst was elucidated employing density functional theory (DFT) simulations. A simple PHIP set‐up was used to generate hyperpolarized [1‐^13^C]fumarate at sufficient ^13^C polarization for *ex vivo* detection of hyperpolarized ^13^C malate metabolized from fumarate in murine liver tissue homogenates, and *in vivo* ^13^C MR spectroscopy and imaging in a murine model of acetaminophen‐induced hepatitis.

## Graphical abstract

**Toward clinical translation**: Hyperpolarized [1‐^13^C]fumarate was prepared by the *trans*‐selective para‐hydrogenation of [1‐^13^C] acetylenedicarboxylate precursor using a [Cp*Ru(MeCN)_3_]PF_6_ catalyst. The feasibility of *in vivo* imaging of necrotic cell death by ^13^C MRI of PHIP‐hyperpolarized exogenously injected fumarate metabolism was demonstrated in an acetaminophen‐induced hepatitis mouse.

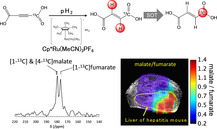

## 1 Introduction

Cell death is a hallmark of various diseases including renal, hepatic and myocardial injury, stroke and Alzheimer's disease, and furthermore plays an important role in cancer development and treatment response.^[1]^ There is an unmet medical need for non‐invasive, quantitative imaging techniques to assess the spatial extent of pathological cell death and thus enable personalization of treatments. While small‐molecule positron emission tomography (PET) probes have been proposed for imaging apoptosis (programmed cell death),^[2]^ the most common type of cell death in various diseases is necrosis (unregulated cell death), for which there are no clinically‐available imaging methods.

The advent of hyperpolarization of ^13^C (or ^15^N) nuclei in biologically‐relevant molecules by dissolution dynamic nuclear polarization (dDNP)^[3]^ has enabled molecular MRI as a means to interrogate metabolism and other cellular processes with an unprecedented sensitivity.[^4^, ^5^, ^6^] In particular, hyperpolarized (HP) [1‐^13^C]pyruvate and its conversion to lactate are exquisitely sensitive to the Warburg effect; regulation of glycolytic metabolism in cancerous cells,[^7^, ^8^, ^9^] with added specificity over ^18^F‐fluorodeoxyglucose (FDG) PET.^[10]^ In recent years, the development of sterile, clinical‐scale dDNP polarizers^[11]^ has enabled the realization of clinical application in humans.[^5^, ^6^, ^12^, ^13^, ^14^] In seminal work, Gallagher and colleagues demonstrated that [1,4‐^13^C_2_]fumarate can also be hyperpolarized by dDNP, and that the metabolic conversion of HP [1,4‐^13^C_2_]fumarate to [1,4‐^13^C_2_]malate can serve as a non‐invasive imaging biomarker for necrotic cell death.^[15]^ The production of HP [1,4‐^13^C_2_]malate has been shown to be a sensitive biomarker to treatment response in tumors,[^15^, ^16^, ^17^] and allow early detection of necrosis due to acute kidney injury^[18]^ and myocardial infarction.^[19]^ HP ^13^C fumarate possesses several desirable properties for application as a necrosis probe *in vivo*; (i) fumarate is an endogenous molecule and is thus biologically safe, (ii) its enzymatic conversion to malate by a fumarase does not require any co‐factors, (iii) exogenously‐delivered HP ^13^C fumarate does not (or very slowly) permeate(s) into healthy cells in which fumarase is present, (iv) upon necrotic loss of cell plasma membrane integrity, fumarase is released into the extracellular space. Thus, in healthy cells no HP ^13^C malate is produced while in necrotic cells HP ^13^C malate is produced in the extracellular space without co‐factors. As such, HP ^13^C malate production is a sensitive marker of cell necrosis.

State‐of‐the‐art dDNP equipment for the production of sterile metabolic imaging probes is costly, and high clinical throughput is constrained by long polarization build‐up times (∼hours).

Parahydrogen‐induced polarization (PHIP)‐wherein the natural spin order of parahydrogen is utilized to generate hyperpolarized ^1^H on a substrate molecule by hydrogenation[^20^, ^21^] and subsequently transfer polarization to a heteronucleus (X=^13^C, ^15^N etc.)^[22]^‐may offer a cost‐effective alternative. Apparatus is relatively cost‐effective and polarization times are rapid (∼seconds)^[23]^ in comparison to dDNP. However, in conventional “hydrogenative” PHIP, the requirement of an unsaturated carbon‐carbon bond on the substrate for hydrogenation constrains the available substrate structure. As a result, until recently, only a small number of biologically‐relevant molecules had been polarized by PHIP for *in vivo* HP ^13^C MRI studies, including; hydroxyethyl [1‐^13^C]propionate (HEP) to probe angiography,[^22^, ^24^, ^25^] and [1‐^13^C]succinate[^26^, ^27^, ^28^] and diethyl [1‐^13^C]succinate,[^26^, ^29^] which are involved in Krebs cycle metabolism. A means to overcome the substrate structure limitation was proposed by Reineri *et al*, who demonstrated that by parahydrogen addition to an unsaturated *ester* precursor of the target molecular probe, spin polarization transfer from ^1^H to the carbonyl ^13^C and subsequent hydrolysis to cleave the “side‐arm” of the ester, the target HP imaging probe can be extracted in the aqueous phase for *in vivo* MR applications. This PHIP side‐arm hydrogenation (PHIP‐SAH) method has enabled the unprecedented generation of HP [1‐^13^C]pyruvate and acetate[^30^, ^31^] and the first *in vivo* studies with this technique.^[32]^

While a PHIP‐SAH‐suitable ester precursor of fumarate is yet to be reported, parallel progress in catalyst chemistry is opening up new avenues for PHIP substrates. The Rh‐based catalysts‐typically of the form [Rh(diene)diphos]+ where diphos is a chelating phosphine‐that are most‐widely used in hydrogenative PHIP experiments are *cis* selective; tending to yield a *cis* isomer as a hydrogenation product. For example, addition of parahydrogen to acetylenedicarboxylate (ADC) yields maleate,[^28^, ^33^] the *cis*‐isomer of butenedioate. Fumarate on the other hand, is the *trans*‐isomer of butenedioate. It was recently demonstrated that commercially available ruthenium‐based catalysts can be used to generate HP [1‐^13^C]fumarate by *trans*‐selective hydrogenation of [1‐^13^C]ADC.[^34^, ^35^] This relies on the *trans*‐stereoselectivity of these ruthenium‐based catalysts[^36^, ^37^] and builds upon early PHIP work on Cp*Ru (Cp*: pentamethylcyclopentadienyl) catalysts.^[38]^ The chemistry is complex and the addition of the reducing agent sodium sulfite is required for ADC hydrogenation to increase the catalyst activity and suppress production of maleate, though the mechanism of sodium sulfite's action is not well understood. A key advantage of this approach for *in vivo* applications is that the reaction can be performed in water. Metabolism of PHIP‐polarized [1‐^13^C]fumarate has now been observed in lysed and healthy tumor cells *in vitro*,[^35^, ^39^] though *in vivo* imaging of cell death has not been reported to date.

In this work, we report; (i) a cost‐effective synthetic pathway for high‐yield production of [1‐^13^C]acetylenedicarboxylic acid, (ii) generation of HP [1‐^13^C]fumarate using a simple hydrogenative PHIP experimental set‐up and the *trans*‐selective Ru‐based catalyst, (iii) elucidation of the *trans*‐selectivity of hydrogenation with the Ru‐based catalyst via density functional theory (DFT) simulations, (iv) *in vitro* magnetic resonance spectroscopy (MRS) of HP ^13^C malate generated from HP [1‐^13^C]fumarate in the mixture with liver homogenate, (v) the feasibility of *in vivo* MRI of PHIP‐polarized [1‐^13^C]fumarate metabolism for detection of cell necrosis in a murine acetaminophen‐induced model of hepatitis, where the anti‐inflammatory agent acetaminophen is well known to cause necrotic cell death in the liver that is often a problem in a clinical practice.

## 2 Results and Discussion

### 2.1 Synthesis of [1‐^13^C]Acetylenedicarboxylic Acid

**Scheme 1:**
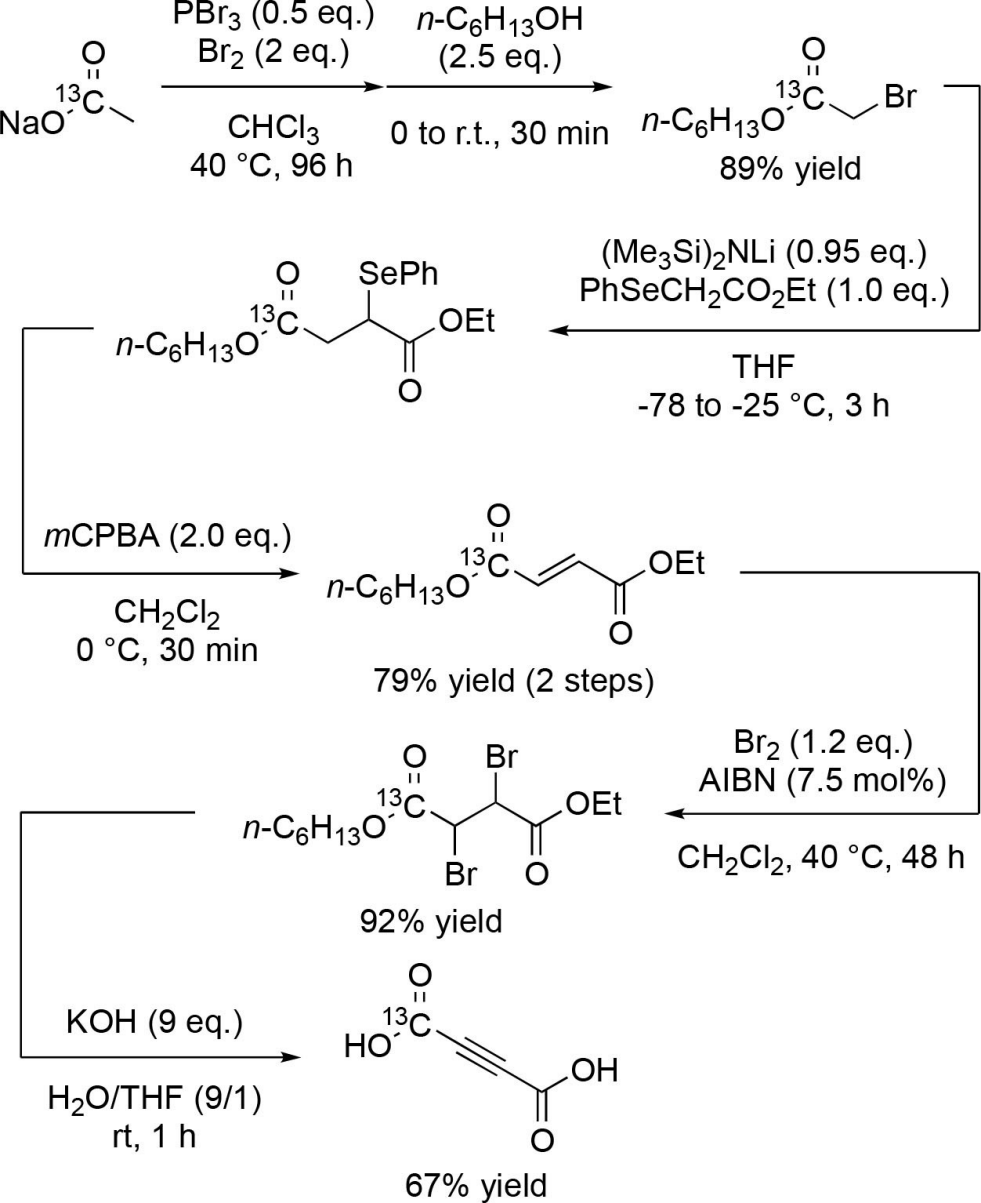
Four‐step synthesis pathway for the [1‐^13^C]fumarate precursor, [1‐^13^C] acetylenedicarboxylic acid, using [1‐^13^C]sodium acetate as a starting material. Full details are provided in the Experimental Section and the Supporting Information.

In general, *in vivo* MRI experiments involving hyperpolarized ^13^C‐labeled tracers consume a much larger amount of the tracer than in most *in vitro* NMR studies. Thus, for hydrogenative PHIP, there is a great need for a low‐cost, high‐yield synthesis pipeline for producing the hydrogenation precursor. Here, this is [1‐^13^C]acetylenedicarboxylic acid, which is available commercially by special order, though costly. We developed a synthesis pathway with [1‐^13^C]sodium acetate as a starting material, as described in Scheme 1, the Experimental Section and Supporting Information. The total yield of the final product was ∼43 %.

### 2.2 DFT Simulations of the Trans‐selective Hydrogenation Reaction of Acetylene Dicarboxylate

**Figure 1:**
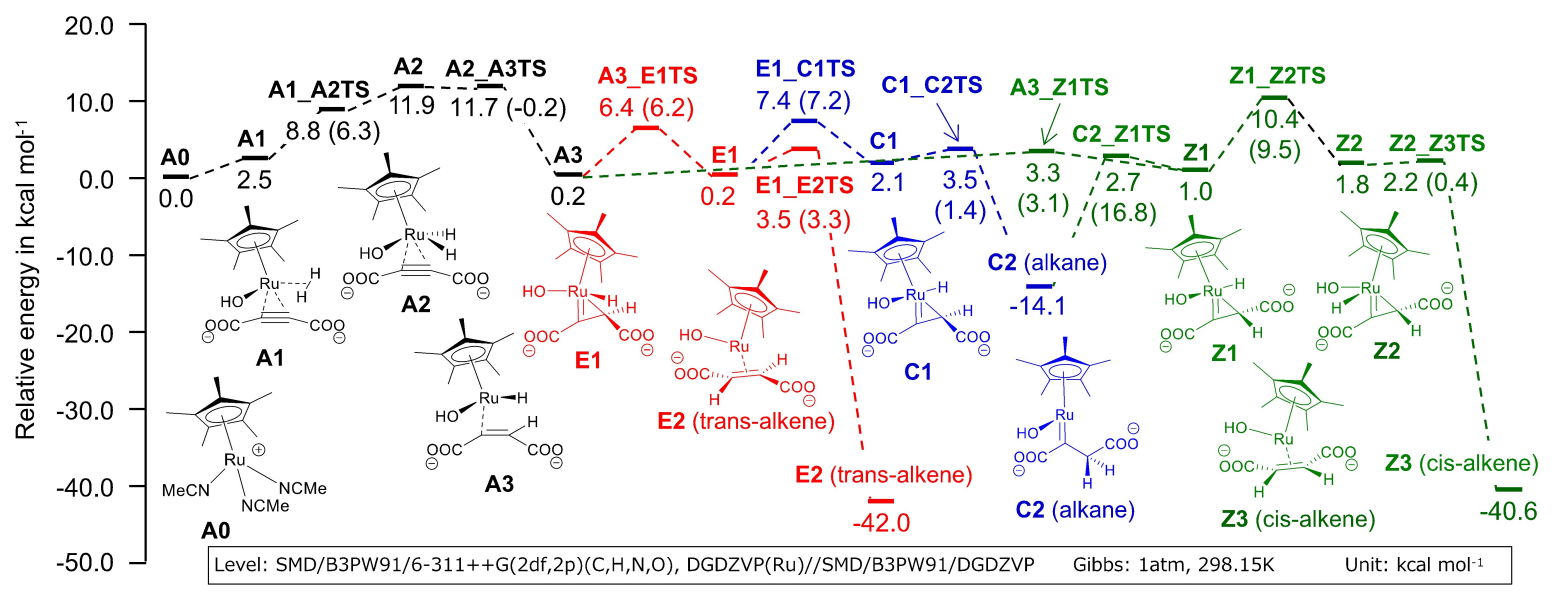
Free energy diagram for the *trans*‐selective hydrogenation reaction of acetylenedicarboxylate using [Cp*Ru(MeCN)_3_]PF_6_ at SMD/B3PW91/6‐311++G(2df,2p)(C,H,N,O),DGDZVP(Ru) //SMD/B3PW91/DGDZVP calculated by DFT simulation. Values in parentheses are the differences of the energies from the previous steps.

M. Leutzsch et al. analyzed the *trans*‐selective hydrogenation of 2‐butyne in CH_2_Cl_2_ using the [Cp*Ru(MeCN)_3_]PF_6_ catalyst using DFT theory.^[37]^ In the following calculation, we referred to the Leutzsch's mechanisms and analyzed the *trans*‐hydrogenation of acetylenedicarboxylate in water using the same catalyst at the SMD/B3PW91/6‐311++G(2df,2p)(C,H,N,O),DGDZVP(Ru)//SMD/B3PW91/DGDZVP level of theory. We show a computed energy diagram under basic conditions in Figure 1. This diagram indicates relative energies for the reaction start point A0. Values in parentheses are activation free energies from the reactant or intermediate of each step in the direction of forward reaction.

**Figure 2:**
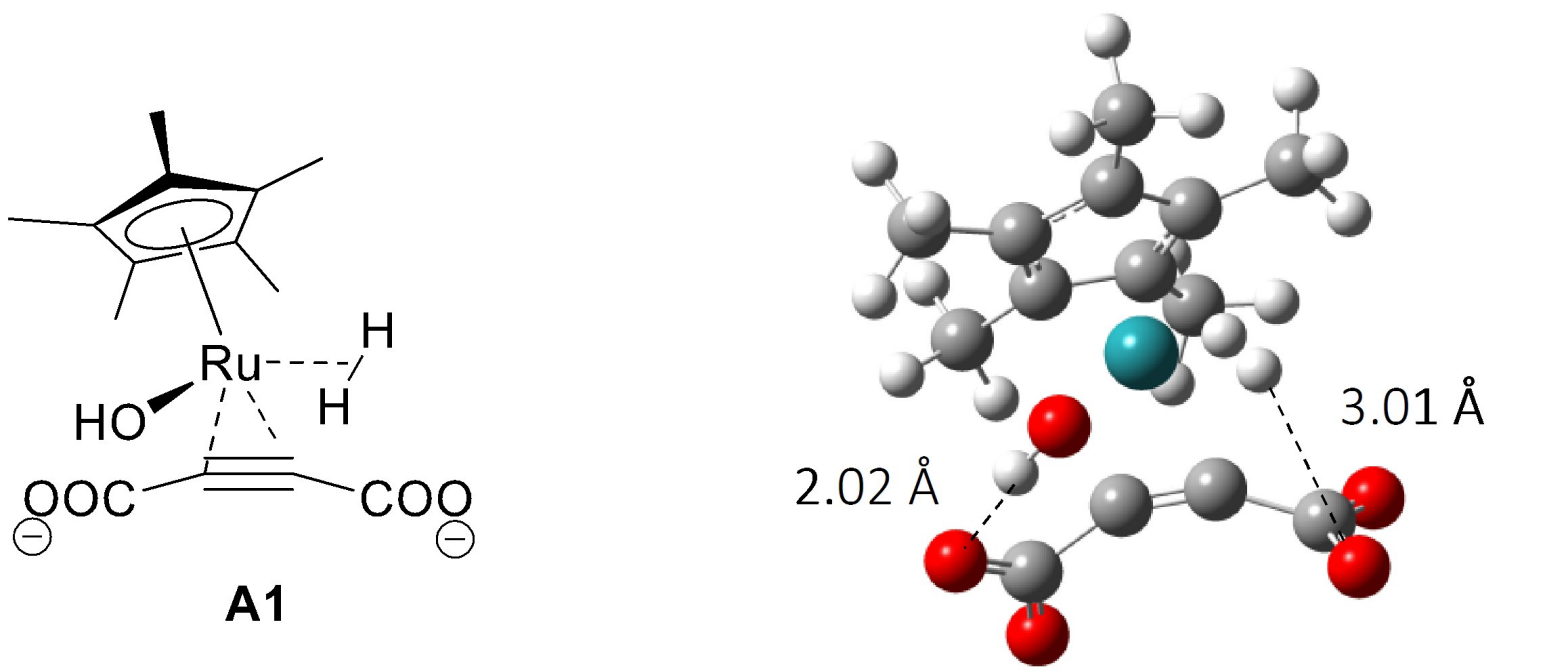
An optimized structure of stable reactant complex of A1.

The first step of this reaction under basic conditions starts from catalyst A0 to A3 via intermediate A1. A1 is a stable complex with coordination of ADC and hydrogen gas shown in Figure 2. ADC coordinates as a dianion and one carboxylate interacts with the proton of the coordinated hydroxide ion by a hydrogen bond of length 2.02 Å. The other carboxylate does not have hydrogen bonds with the catalyst. A1 proceeds to an active complex A2 and further to A3 with the transfer of a hydrogen atom to the triple bond. The activation energy of the step from A0 to A3 was computed as ΔG^≠^=11.9 kcal mol^−1^. After the formation of A3, the reaction proceeds to Z1 and turns over and reach C2 (alkane) via a transition state C2_Z1TS without any notable barrier. From C2, there are two reaction routes. One is a route to trans‐alkene E2 via C1 and E1 and the other is a route to cis‐alkene Z3 via Z1 and Z2. By considering these routes as pseudo‐one‐step reactions (as intermediates C1 and Z1 are not stable), the rate determining ΔG ^≠^ of these routes are 21.5 and 24.5 kcal mol^−1^ at the transition states of E1_C1TS and Z1_Z2TS, respectively. Based on Eyring's absolute reaction rates theory, the difference of activation free energies ΔΔG^≠^=3.0 kcal mol^−1^ gives a production ratio of trans and cis alkene at 80 °C of 98.6 : 1.4. Though the actual production ratio will change depending on the concentration of substrate and catalyst, energies of recovery cycles of the catalyst, pH, the diffusion of hydrogen bubbles and so on, this simulation suggests that the formation of trans‐alkene is dominant in this system and supports observed experimental results.

**Figure 3:**
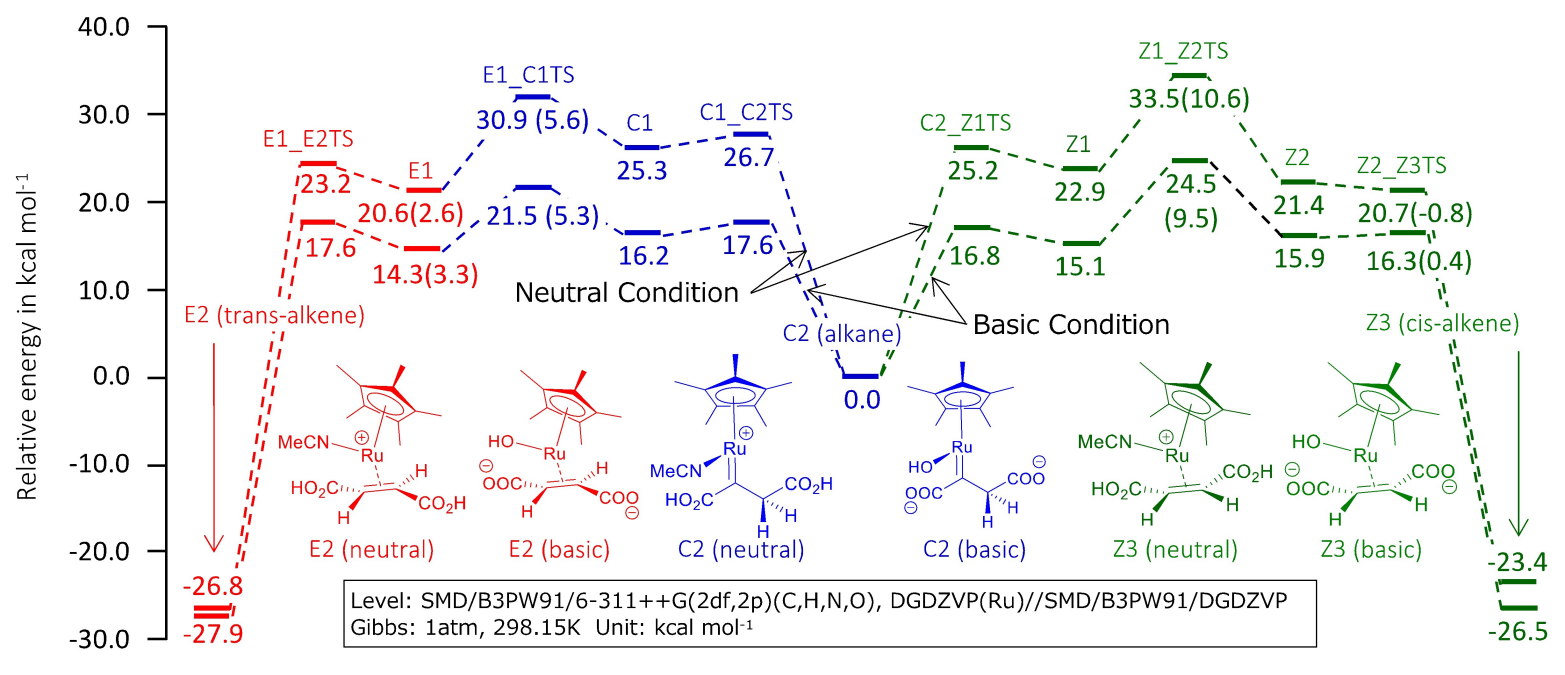
Comparative free energy diagram for the hydrogenation of acetylenedicarboxylic acid using [Cp*Ru(MeCN)_3_]PF_6_ in basic and neutral conditions from C2. Values in parentheses are the differences of the energies from the previous steps.

Under neutral conditions, the reaction proceeds with the same mechanism as that of basic conditions and produces a semi‐stable C2. Figure 3 shows the free energy diagrams leading to trans‐alkene E2 and cis‐alkene Z3 from C2 in neutral and basic conditions comparatively. The rate determining ΔG^≠^ of the routes to E2 and Z3 in neutral conditions were computed as 30.9 and 33.5 kcal mol^−1^. ΔΔG^≠^=2.6 kcal mol^−1^ is smaller than the basic condition and it causes slight deterioration of the selectivity. The production ratio in this condition at 80 °C can be calculated as trans : cis=97.6 : 2.4. We can also calculate the difference between basic and neutral reaction rates by the Eyring's equation. At 80 °C, normalizing to the reaction rate in basic conditions (1.0), the relative rate in neutral conditions is calculated as 1.5×10^−6^ to produce trans‐alkene E2 and 2.7×10^−6^ to cis‐alkene Z3. Therefore, the reaction in neutral conditions is orders of magnitude slower than that in basic conditions, and thus considered difficult to proceed in the same reaction time.

### 2.3 Parahydrogen Addition Reaction

[1‐^13^C]fumarate concentrations of the order of 25 mM (hydrogenation percentage ∼10 %) were obtained using the procedures described in the Experimental Section. This is in approximate agreement with that of Knecht et al who employed an automated hydrogenation system (for a ∼10 s hydrogenation time; see Figure 2a of Ref. [39]). Crucially, this concentration of fumarate is comparable to that used in the original demonstration of *in vivo* MR imaging of necrosis using dDNP‐polarized fumarate (though in that study, doubly‐labeled [1,4‐^13^C]fumarate was used, which leads to double the NMR sensitivity for a given concentration).^[15]^ Nevertheless, the achievable [1‐^13^C]fumarate concentration remains a major limitation of our approach. The advent of fully‐automated apparatus and reaction chambers capable of withstanding gas pressures exceeding 10 bar should lead to more efficient hydrogenation and thus increased concentration and/or polarization of [1‐^13^C]fumarate doses for *in vivo* applications in the near future. While automated set‐ups are of course desirable, the manual procedure implemented here has advantages in terms of minimization of the fluid path length and in‐turn potential losses of substrate volume or polarization (e. g. through contact with paramagnetic impurities).

### 2.4 Spin Order Transfer

*Trans*‐selective hydrogenation of [1‐^13^C]ADC with the ruthenium‐based catalyst Cp*Ru(MeCN)_3_PF_6_ results in the production of [1‐^13^C]fumarate with two parahydrogen‐derived hyperpolarized ^1^H nuclei. Efficiency of spin order transfer from these hyperpolarized ^1^H to the labeled ^13^C spin was investigated by applying either INEPT‐type spin order pulse sequences L‐PH‐INEPT+ or S2hM using nested ^1^H and ^13^C RF coils in a 1.5T MRI scanner, or a magnetic field cycling (MFC) approach using a zero‐field chamber. More than 12 % (12 +/−2 % over 4 measurements) ^13^C polarization at the time of the subsequent ^13^C NMR/MRI scan was achieved by MFC and S2hM with the Cp*Ru(MeCN)_3_PF_6_ catalyst. While this polarization value is reasonable, we believe there is still room for improvement. In particular, Eills et al. recently reported 24 % ^13^C‐polarization of fumarate by applying a constant adiabaticity magnetic field cycle^[35]^ and further improvement to >30 % by a new purification procedure,^[39]^ but we have not yet succeeded to reproduce their experiments. On the other hand, Korchak et al. reported the feasibility of 20 % ^13^C‐polarization of acetate from vinyl acetate as a precursor by using another INEPT‐type SOP sequence known as ESOTHERIC.^[40]^ Based on the J‐coupling network, fumarate should be more efficiently polarized than acetate. Collectively, it seems too early to conclude whether MFC‐ or INEPT‐type approaches are more efficient for polarizing fumarate from our still primitive experimental setup.

### 2.5 *In vitro* MRS of [1‐^13^C]Fumarate in Tissue Homogenates

**Figure 4:**
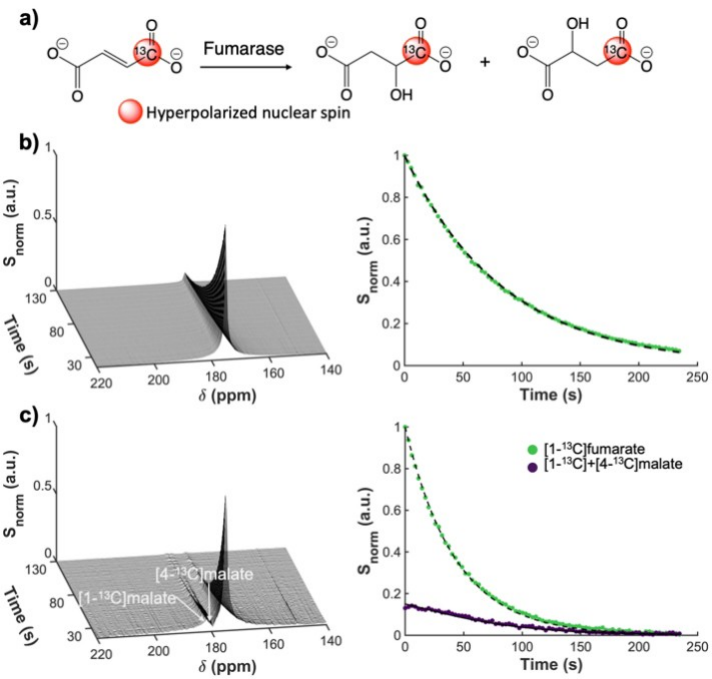
a) Schematic of the conversion of [1‐^13^C]fumarate into [1‐^13^C] and [4‐^13^C]malate by fumarase. b) Dynamic ^13^C NMR spectra of hyperpolarized [1‐^13^C]fumarate at 176 ppm produced by MFC‐based PHIP (left) and the fitted signal decay (right). c) Generation of hyperpolarized [1‐^13^C] and [4‐^13^C]malate at around 181 ppm by mixing hyperpolarized [1‐^13^C]fumarate with mouse liver homogenates *in vitro* (left) and signal kinetics of the [1‐^13^C]fumarate and [1‐^13^C] and [4‐^13^C]malate peaks fitted with a two‐compartment exchange model (yielding T_1_ ∼80 s for fumarate) (right). Note: the “DC offset” at the beginning of the purple curve suggests that considerable metabolic exchange occurred prior to the acquisition; this prevented us from obtaining a realistic fitted estimate for the fumarate‐malate conversion rate.

In dDNP studies, hyperpolarized [1,4‐^13^C_2_]fumarate has been successfully used to detect necrotic cell death in various disease

models. To investigate whether hyperpolarized [1‐^13^C]fumarate prepared by PHIP works comparably as a cell death imaging probe, hyperpolarized [1‐^13^C]fumarate solution was mixed with mouse liver homogenates. Time dependent generation of the typical doublet peak of [1‐^13^C] and [4‐^13^C]malate, whose production itself is a biomarker of necrotic cell membrane destruction, was observed at 181 ppm immediately after infusion of HP [1‐^13^C]fumarate (Figure 4). By virtue of the fact that the enzymatic activity of fumarase does not require any cofactors, the [1‐^13^C] and [4‐^13^C]malate signal lasted for up to several minutes and became undetectable at a similar time‐point to that of the [1‐^13^C]fumarate peak.

### 2.6 *In vivo* Imaging of [1‐^13^C]fumarate Metabolism

**Figure 5:**
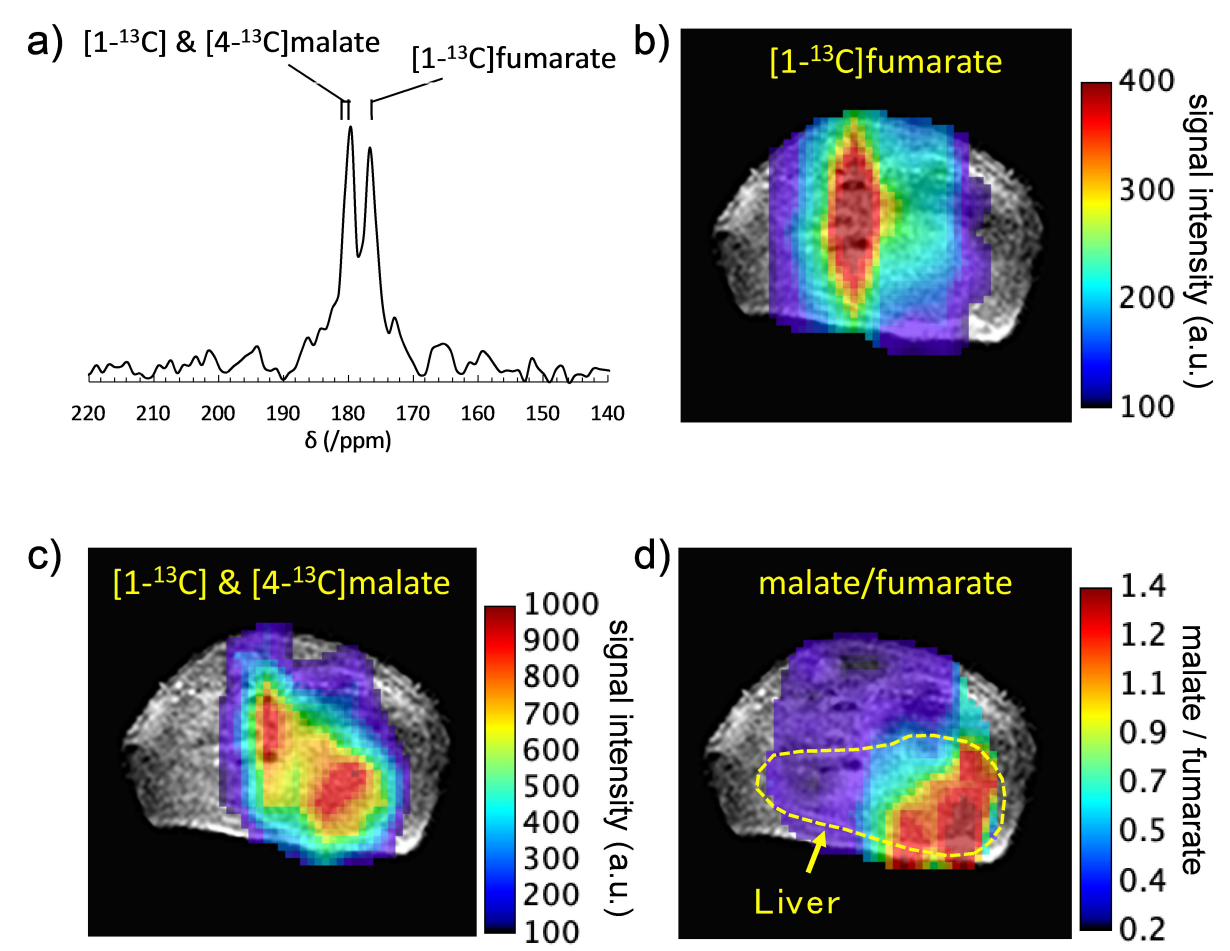
*In vivo* CSI of hyperpolarized [1‐^13^C]fumarate metabolism in an acetaminophen‐induced hepatitis mouse. a) Representative ^13^C NMR spectrum of hyperpolarized [1‐^13^C]fumarate and its metabolite for a CSI pixel at the liver. b) Map of hyperpolarized [1‐^13^C]fumarate CSI signal intensity overlaid on an anatomical ^1^H MRI image. c) Map of hyperpolarized [1‐^13^C] and [4‐^13^C]malate. d) parametric map of the malate/fumarate ratio; a biomarker of cellular necrosis.

The feasibility of *in vivo* cell death imaging using PHIP‐polarized [1‐^13^C]fumarate was assessed using an acetaminophen‐induced hepatitis mouse model. Two‐dimensional chemical shift imaging (CSI) performed at 4 hours after intraperitoneal injection of acetaminophen on a 1.5T MRI scanner showed the production of hyperpolarized [1‐^13^C] and [4‐^13^C]malate in the liver region (Figure 5a). Note: the [1‐^13^C] and [4‐^13^C]malate peaks were inseparable *in vivo* (though sometimes observable as a shoulder bump), because their 1.2 ppm chemical shift difference is much smaller than the typical ^13^C spectral linewidth *in vivo* in our permanent magnet‐based MRI scanner (over 2 ppm). The distribution of hyperpolarized [1‐^13^C]fumarate at ∼20 sec after injection showed strong signal from the abdominal aorta (Figure 5b), whereas that of [1‐^13^C] and [4‐^13^C]malate signal exhibited localization in the liver. High signal intensity around the abdominal aorta on the malate map may result from contamination by the much more intense [1‐^13^C]fumarate peak. By normalizing the signal intensity of the malate peak to that of the fumarate peak, a parametric map of the malate‐to‐fumarate ratio was produced that more clearly showed the localized production of the cell death marker malate in the liver with acetaminophen‐indued hepatitis.

In this *in vivo* experiment, the [Cp*Ru(CH_3_CN)_3_]PF_6_ catalyst was removed by a metal trapping column filling a QuadraPure® TU (Sigma‐Aldrich Co. LLC.) (see Supporting Information), however some unreacted ADC still remained in the injection solution. Although we did not see any cytotoxic effects of ADC in these preliminary *in vivo* experiments, complete removal of ADC is desirable for future studies. We note that Knecht et al recently reported a promising fumarate purification method based on precipitation as a solid in concentrated HCl, however this method relies on the addition of concentrated fumarate to encourage precipitation and thus may not be practicable for *in vivo* application at present.^[39]^

## 3 Conclusions

With low‐cost polarization hardware and rapid polarization times, hydrogenative PHIP with Ru‐based catalysts holds great promise as a scalable route to clinical use of HP [1‐^13^C]fumarate as a probe of cellular necrosis. In this work, we have investigated several aspects of the process of PHIP‐induced polarization of [1‐^13^C]fumarate and its application *in vitro* and *in vivo*. In particular, we have reported a cost‐effective synthetic pathway for high‐yield production [1‐^13^C]acetylenedicarboxylic acid that should help facilitate dissemination of the technique. In addition, we have demonstrated initial feasibility of *in vivo* MRI of PHIP‐polarized [1‐^13^C]fumarate metabolism for detection of cell necrosis in a mouse model of hepatitis. These findings should facilitate further *pre*‐*clinical* studies and represent key first steps in the eventual clinical translation.

## Experimental Section

### Synthesis of [1‐^13^C]Acetylenedicarboxylic Acid

Full details of the synthesis of the [1‐^13^C]fumarate precursor, [1‐^13^C]acetylenedicarboxylic acid, along with NMR spectra of the reaction intermediates and final product, are provided as Supporting Information. Briefly, the following describes the four‐step synthesis procedure with [1‐^13^C]sodium acetate as a starting material that was followed in this work (see also Scheme 1).

(i): Sodium acetate‐1‐^13^C (1.66 g, 1 Eq, 20.0 mmol) was melted, combined with CHCl_3_ (6.6 mL), PBr_3_ (2.71 g, 943 μL, 0.5 Eq, 10.0 mmol) and bromine (6.39 g, 2.06 mL, 2 Eq, 40.0 mmol) and warmed to 55 °C. After stirring for 96 h at 55 °C, the reactants were cooled to 0 °C and hexan‐1‐ol (5.11 g, 6.28 mL, 2.5 Eq, 50.0 mmol) was added. The reactants were then stirred for 30 min at 25 °C, poured into a solution of 1 M aqueous Na_2_SO_3_ and extracted with hexane. The organic layers were washed with saturated NaHCO_3_, dried over Na_2_SO_4_ and evaporated under vacuum. The residue was purified by column chromatography on silica gel eluting with 0∼60 % CH_2_Cl_2_ in hexane to give hexyl 2‐bromoacetate‐1‐^13^C (4.00 g, 17.8 mmol, 89 %) as a colorless oil.

(ii): To a stirred solution of HMDS (1.01 g, 1.30 mL, 1 Eq, 6.25 mmol) in THF (20 mL) was added n‐BuLi (380 mg, 2.12 mL, 2.80 molar, 0.95 Eq, 5.93 mmol) at 0 °C under argon atmosphere. After stirring for 10 min, the solution was cooled to −78 °C and ethyl 2‐(phenylselanyl)acetate (1.52 g, 1 Eq, 6.25 mmol) was added. After stirring for 30 min at −78 °C, hexyl 2‐bromoacetate‐1‐^13^C (1.40 g, 1 Eq, 6.25 mmol) was added and the reactants were warmed to −25 °C over 3 h and quenched with saturated aqueous NH_4_Cl. The organic layer was extracted with hexane, dried over Na_2_SO_4_ and evaporated under vacuum. To the residue containing 1‐ethyl 4‐hexyl 2‐(phenylselanyl)succinate‐4‐^13^C, CH_2_Cl_2_ (10 mL) was added. After cooling to 0 °C, mCPBA (3.32 g, 65 % Wt, 2 Eq, 12.5 mmol) was added and the reactants were stirred for 30 min at 0 °C, diluted with hexane (50 mL) and filtrated through a pad of Celite. The filtrate was evaporated under vacuum and the residue was purified by column chromatography on silica gel eluting with AcOEt/hexane 0 to 4 % to give ethyl hexyl fumarate‐4‐^13^C (1.13 g, 4.93 mmol, 79 %) as a pale‐yellow oil.

(iii): Ethyl hexyl fumarate‐4‐^13^C (1.13 g, 1 Eq, 4.93 mmol) in CH_2_Cl_2_ (5 mL), AIBN (40.5 mg, 0.05 Eq, 246 μmol) and Br_2_ (945 mg, 305 μL, 1.2 Eq, 5.91 mmol) were combined in a flask and warmed to 40 °C. After stirring for 24 h, the progress of the reaction was monitored by TLC (Hex/CH_2_Cl_2_ 1/1). AIBN (20.3 mg, 0.025 Eq, 123 μmol) was added to the flask and the reaction was stirred for 24 h at 40 °C. The reaction was quenched with 1 M aqueous Na_2_SO_3_, extracted with CH_2_Cl_2_, dried over Na_2_SO_4_ and evaporated under vacuum. The residue was purified by column chromatography on silica gel eluting with CH_2_Cl_2_/hexane 5 to 50 % to give 1‐ethyl 4‐hexyl 2,3‐dibromosuccinate‐4‐^13^C (1.77 g, 4.54 mmol, 92 %, dr=83 : 17) as a colorless oil.

(iv): To a test tube containing KOH (2.52 g, 85 % Wt, 9 Eq, 38.2 mmol) dissolved in water (3.8 mL) at 0 °C, 1‐ethyl 4‐hexyl 2,3‐dibromosuccinate‐4‐^13^C (1.65 g, 1 Eq, 4.24 mmol) dissolved in THF (0.4 mL) was added and the reactants were stirred vigorously at 0 °C for 2 h. The solution was washed with hexane and the aqueous phase was acidified with 12 N HCl (4 mL). The solution was washed with toluene to remove impurities. The organic materials were then extracted with AcOEt. The combined organic layers were dried over Na_2_SO_4_ and evaporated under vacuum to give [1‐^13^C]acetylenedicarboxylic acid (328 mg, 2.85 mmol, 67.2 %, ∼90 % purity as determined by ^13^C NMR).

### DFT Simulations of Cp*Ru‐catalyzed trans‐Hydrogenation

It is known that a Cp*Ru‐catalyzed *trans*‐hydrogenation of acetylenedicarboxylic acid in water is accelerated and the *trans*‐selectivity of the product is improved under basic conditions.^[34]^ To clarify this phenomenon, density functional theory calculations were carried out and reaction mechanisms were analyzed. B3PW91[^41^, ^42^] functional and basis sets of 6‐311G(d,p) and DGDZVP[^43^, ^44^] for H, C, N, O atoms and Ru atom respectively were applied for energies, and the DGDZVP basis set was applied for optimizing geometries. The self‐consistent reaction field (SCRF) method with the Solvation Model based on Density (SMD) model^[45]^ was used to describe the aqueous solvent. As substrate complexes for reactants and products, we searched for and adopted structures that seemed to be the most stable. We searched transition states structures with using the energy gradient method and confirmed that they had only one imaginary frequency. In order to confirm whether the transition states lead to products and reactants, intrinsic reaction coordinate (IRC) calculations^[46]^ were performed. Vibration analyses at 298.15 K and 1 atm were executed for all structures to calculate Gibbs correlation energies, hence reactions were evaluated using relative Gibbs free energy. All calculations were performed using Gaussian09.^[47]^

### Parahydrogen Generation

Parahydrogen gas was generated using a home‐built system based on that described in reference.^[48]^ Briefly, hydrogen gas was flowed at 1–2 NL/min through a quarter inch copper tube containing iron(III) oxide (371254‐250G, Sigma‐Aldrich, St Louis, MO) catalyst wound on the 2^nd^ stage of a Gifford‐McMahon cryocooler (Sumitomo Heavy Industries, Ltd., Tokyo, Japan) at 15–25 K and housed within a cryostat‐vacuum system (Thermal Block Company, Saitama, Japan). Over time, the converted parahydrogen gas was accumulated in a stainless‐steel cylinder (either 316 L‐HDF4‐500, 500 cm^3^ or 304 L‐HDF4‐1GAL, 3785 cm^3^, Swagelok, Solon, OH) until a pressure of 9–10 bar was obtained. This system reproducibly yields a parahydrogen concentration of 90–95 % with a lifetime of the order of weeks in the storage cylinders, as quantified by a benchtop Raman spectrometer (EZRaman, TSI Incorporated, MN) with a 520 nm laser.

### Parahydrogen Addition Reaction

Samples were prepared similarly to as described in references.[^34^, ^35^, ^39^] Briefly, 3.78 mg of sodium sulfite (final 50 mM) was added to a high‐pressure glass vial and introduced into a glovebox under argon atmosphere (<0.5 % O_2_), and mixed with 596 μL D_2_O and 4 mg of [RuCp*(MeCN)_3_]PF_6_ (final 13.2 mM, #667412, Sigma‐Aldrich, St Louis, MO). The sample was then subjected to sonication for up to 60 min, until the catalyst was completely dissolved (temperature was gradually increased to 30–40 °C by this process). 3.45 mg of [1‐^13^C]acetylenedicarboxylic acid (final 50 mM) was added to the catalyst solution, along with 4 μL of 40 % NaOD. The pH of the final solution was around 10.

The reaction vial was then removed from the glovebox and heated for ∼90 sec on a thermal block at 90 °C. The vial cap was replaced with a custom cap connected to 1/16’’ OD PEEK tubing. Parahydrogen gas was injected at 8–10 bar, and the vial was manually shaken for the duration of the parahydrogen exposure (either 10 sec or 60 sec), after which the gas source was closed, and the gas pressure released prior to opening the vial.

After experiments, the reaction vials were rinsed with chloroform and washed with 1 M HCl and 50 mM EDTA heated to 70 °C to remove residual catalyst and other impurities. In a small subset of samples that were not used for subsequent spin order transfer for *in vitro* or *in vivo* ^13^C MR studies, the percentage of hydrogenation was quantified by ^1^H NMR using a JEOL ECS400 C (Delta V5.0.4) 400 MHz NMR spectrometer.

### Spin Order Transfer

Spin order transfer from ^1^H to ^13^C was induced using one of several different methods, including magnetic field cycling (MFC)[^49^, ^50^] and RF pulse sequences; specifically, an INEPT‐type RF pulse sequence adapted for parahydrogen applications; longitudinal (l)‐PH‐INEPT +,[^51^, ^52^] and the recently‐developed Singlet to heteronuclear Magnetisation (S2hM) sequence.^[53]^ L‐PH‐INEPT+ and S2hM were implemented on a home‐built 1.5 T permanent magnet MRI system with a Japan REDOX spectrometer (Japan REDOX Ltd., Fukuoka, Japan), with timings determined from the J‐couplings in [1‐^13^C]fumarate, assuming an AA'X system. Home‐built ^1^H and ^13^C RF coils of saddle or solenoid design were nested and utilized to play out the RF sequences while a vial or syringe containing ^1^H‐hyperpolarized [1‐^13^C]fumarate was placed inside the coil active region (immediately after the parahydrogenation reaction).

In MFC experiments, the vial containing ^1^H‐hyperpolarized [1‐^13^C]fumarate after parahydrogen addition was rapidly inserted into a zero‐field chamber and either (i): (adiabatically) lifted out over the course of a few seconds, (“manual”) approach; or (ii) subjected to an electronically‐controlled magnetic field sweep of bi‐linear or exponential time profile that adiabatically increased the field from an initial zero field, (“automated”) approach. Both methods induced sufficient spin order transfer from ^1^H to ^13^C. The zero‐field chamber comprised three μ‐metal cylinders (ZG‐206, Magnetic Shield Corporation, Bensenville, IL) and was calibrated using a three‐axis magnetic field sensor (Mag 690–100, Bartington Instruments, Oxon, UK) and three orthogonal field coils (an additional coil was used for the electronic field sweep). Timings of the diabatic field demagnetization and adiabatic remagnetization were optimized by a combination of empirical experiments and density matrix simulations based on the heteronuclear couplings between the [1‐^13^C] and parahydrogen nuclei in fumarate.[^49^, ^54^]

### *In vivo* Chemical Shift Imaging of [1‐^13^C]Fumarate Metabolism

#### Ethical Statement for Animal Experiments

All animal experiments were performed under the ‘Law for The Care and Welfare of Animals in Japan′ and were approved by the Animal Experiment Committee of Hokkaido University (Approval No.16‐0058).

#### In vitro Tissue Homogenate Studies

MR spectroscopy was performed on either a 60 MHz benchtop NMR system (NMReady‐60 Pro, Nanalysis Corp., Canada), or a home‐built 1.5 T permanent magnet MRI system with a multinuclear spectrometer (Medalist, Japan REDOX Ltd). *In vitro* spectroscopic acquisitions involved the dynamic acquisition of ^13^C spectra over a period of 3–5 minutes until the [1‐^13^C]fumarate signal became undetectable. Typical parameters were as follows: TR 3 s; flip angle ∼20 °; spectral bandwidth ∼200 ppm, centred on the [1‐^13^C]fumarate resonance.

Liver tissue derived from healthy mice was homogenized and placed into a standard NMR tube (NMReady system), or small chemical vial (MRI system). HP [1‐^13^C]fumarate was infused into the homogenate mixture by injecting through 1/16’’ OD PTFE or PEEK tubing from a syringe outside the magnet bore. Because it is necessary to perform hydrogenation of [1‐^13^C]ADC under basic conditions, neutralization was required prior to injection into homogenate solution or animals.

#### In vivo Animal Studies

Female C3H/HeJYokSlc mice were obtained from Japan SLC Inc. (Shizuoka, Japan) and instilled intraperitoneally with acetaminophen (200 mg/kg body weight) to induce a model of hepatitis. MR spectroscopic imaging was performed at 4–6 hours after administration of acetaminophen. The 1.5 T permanent magnet MRI system with a Japan REDOX spectrometer was used in combination with home‐built RF coils which consists of an inside solenoid coil for ^13^C and an outside saddle coil for ^1^H channels. *In vivo* imaging experiments were performed using a conventional 2D spatially phase‐encoded ^13^C chemical shift imaging (CSI) pulse sequence. Up to 4 dynamic CSI datasets were acquired over a period of 2 minutes. Typical parameters were set as follows: 16×16 matrix; FOV 32×32 mm (in‐plane spatial resolution 2×2 mm); slice thickness 20 mm; TE/TR, 10/75 ms; flip angle ∼20°; spectral bandwidth, 2 kHz. HP [1‐^13^C]fumarate was injected through the tail vein over the course of 10 seconds, using a plastic cannula connected to a syringe outside the magnet bore via 1/16’’ OD PTFE or PEEK tubing. Immediately prior to injection, the [CpRu(CH_3_CN)_3_]PF_6_ catalyst was removed by a column filled with a QuadraPure® TU (Sigma‐Aldrich Co. LLC.) metal scavenger resin (However, we note that some unreacted ADC remained in the injected solution).

## Supporting information

As a service to our authors and readers, this journal provides supporting information supplied by the authors. Such materials are peer reviewed and may be re‐organized for online delivery, but are not copy‐edited or typeset. Technical support issues arising from supporting information (other than missing files) should be addressed to the authors.

Supplementary
