# Supplementary material for: Hyperpolarized 13C Magnetic Resonance Imaging of Fumarate Metabolism by Parahydrogen‐induced Polarization: A Proof‐of‐Concept in vivo Study
Source: Chemphyschem. 2021 Mar 18;22(10):915–23. doi: 10.1002/cphc.202001038 (PMC8251594; doi:10.1002/cphc.202001038)
Supplement: Supplementary file 1 — Supplementary [file CPHC-22-915-s001.pdf]

ChemPhysChem

Supporting Information

Hyperpolarized ^{13}C Magnetic Resonance Imaging of Fumarate Metabolism by Parahydrogen-induced Polarization: A Proof-of-Concept *in vivo* Study

Neil J. Stewart, Hitomi Nakano, Shuto Sugai, Mitsushi Tomohiro, Yuki Kase, Yoshiki Uchio, Toru Yamaguchi, Yujirou Matsuo, Tatsuya Naganuma, Norihiko Takeda, Ikuya Nishimura, Hiroshi Hirata, Takuya Hashimoto,* and Shingo Matsumoto*

Supporting Information

S-1. Synthesis of [1-^{13}C]acetylenedicarboxylic acid

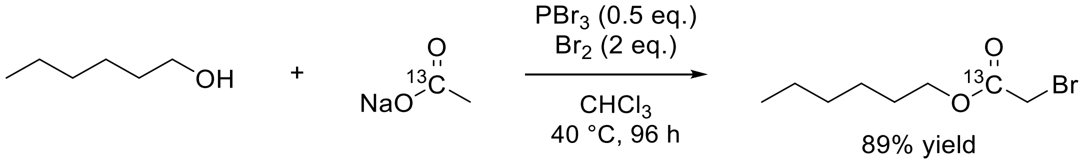

Sodium acetate-1-^{13}C (1.66 g, 1 Eq, 20.0 mmol) was added to a round-bottom flask equipped with a three-necked joint and flame-dried under vacuum till the material melts. The flask was purged with argon and filled with $CHCl_3$ (6.6 mL). To this suspension were added PBr_3 (2.71 g, 943 µL, 0.5 Eq, 10.0 mmol) and bromine (6.39 g, 2.06 mL, 2 Eq, 40.0 mmol). The reaction was warmed to 55 °C and the three-necked joint was replaced with a glass stopper.

(*Caution: set up the reaction behind explosion-proof shield in a well-ventilated fume hood.*)

After stirring for 96 h at 55 °C, the reaction was cooled to 0 °C and hexan-1-ol (5.11 g, 6.28 mL, 2.5 Eq, 50.0 mmol) was added to the solution. The reaction was then stirred for additional 30 min at 25 °C, poured into a solution of 1M aqueous Na_2SO_3 and extracted with hexane. The combined organic layers were washed with saturated $NaHCO_3$, dried over Na_2SO_4 and evaporated under vacuum. The residue was purified by column chromatography on silica gel eluting with 0~60% CH_2Cl_2 in hexane to give hexyl 2-bromoacetate-1-^{13}C (4.00 g, 17.8 mmol, 89%) as a colorless oil.

1H-NMR (396 MHz, CHLOROFORM-D) δ 4.15 (td, J = 6.7, J(^{13}C-H) = 3.0 Hz, 2H), 3.81 (d, J(^{13}C-H) = 4.8 Hz, 2H), 1.59-1.68 (m, 2H), 1.24-1.38 (m, 6H), 0.87 (t, J = 7.0 Hz, 3H).

13C-NMR (100 MHz, CHLOROFORM-D) δ 171.35, 167.43, 66.56, 31.43, 28.44 (d, J = 1.9 Hz), 26.05 (d, J = 64.8 Hz), 25.49, 22.74, 22.58, 14.06

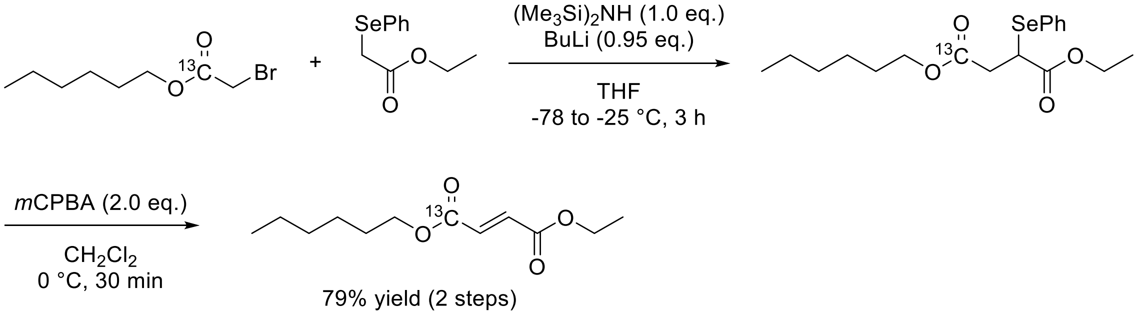

To a stirred solution of HMDS (1.01 g, 1.30 mL, 1 Eq, 6.25 mmol) in THF (20 mL) was added nBuLi (380 mg, 2.12 mL, 2.80 molar, 0.95 Eq, 5.93 mmol) at 0 °C under argon atmosphere. After stirring for 10 min, the solution was cooled to -78 °C. Ethyl 2-(phenylselanyl)acetate (1.52 g, 1 Eq, 6.25 mmol) was added to this solution at the same temperature. After stirring for 30 min at -78 °C, hexyl 2-bromoacetate-1-^{13}C (1.40 g, 1 Eq, 6.25 mmol) was added to this mixture. The reaction was gradually warmed to -25 °C over 3 h and quenched with saturated aqueous NH_4Cl. The organic layer was extracted with hexane, dried over Na_2SO_4 and evaporated under vacuum. The residue was used without further purification.

To the residue containing 1-ethyl 4-hexyl 2-(phenylselanyl)succinate-4-^{13}C was added CH_2Cl_2 (10 mL). After cooling to 0 °C. *m*CPBA (3.32 g, 65% Wt, 2 Eq, 12.5 mmol) was added to this solution and the reaction was stirred for 30 min at 0 °C (white precipitate). The reaction was diluted with hexane (50 mL) and filtrated through a pad of Celite. The filtrate was evaporated under vacuum and the residue was purified by column chromatography on silica gel eluting with AcOEt/hexane 0 to 4% to give ethyl hexyl fumarate-4-^{13}C (1.13 g, 4.93 mmol, 79%) as pale yellow oil.

1H-NMR (396 MHz, CHLOROFORM-D) δ 6.78-6.86 (m, 2H), 4.24 (q, J = 7.2 Hz, 2H), 4.17 (td, J = 6.6, J(^{13}C-H) = 2.8 Hz, 2H), 1.62-1.69 (m, 2H), 1.28-1.39 (m, 9H), 0.87 (t, J = 6.7 Hz, 3H).

13C-NMR (100 MHz, CHLOROFORM-D) δ 167.43, 165.17, 133.73 (d, J = 74.7 Hz), 133.67, 65.60, 61.41, 31.46, 28.54, 25.62, 22.59, 14.19, 14.06.

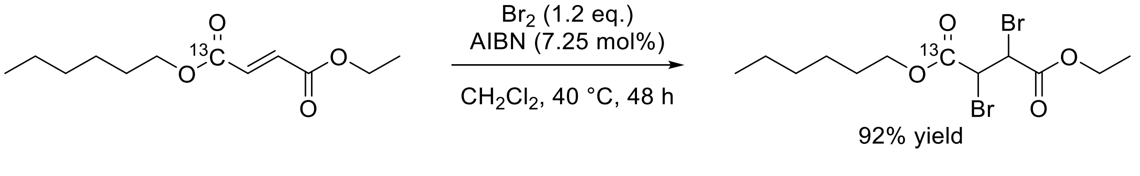

To a flask equipped with a three-necked joint filled with argon gas were added ethyl hexyl fumarate-4-^{13}C (1.13 g, 1 Eq, 4.93 mmol) in CH_2Cl_2 (5 mL), AIBN (40.5 mg, 0.05 Eq, 246 μmol) and Br_2 (945 mg, 305 μL, 1.2 Eq, 5.91 mmol). The solution was warmed to 40 °C and the three-necked joint was replaced with a glass stopper. After stirring for 24 h, progress of the reaction was monitored by TLC (Hex/CH_2Cl_2 1/1). AIBN (20.3 mg, 0.025 Eq, 123 μmol) was added to the flask and the

reaction was stirred for additional 24 h at 40 °C. The reaction was quenched with 1M aqueous Na_2SO_3, extracted with CH_2Cl_2, dried over Na_2SO_4 and evaporated under vacuum. The residue was purified by column chromatography on silica gel eluting with CH_2Cl_2/hexane 5 to 50% to give 1-ethyl 4-hexyl 2,3-dibromosuccinate-4-^{13}C (1.77 g, 4.54 mmol, 92 %, dr = 83:17) as colorless oil.

1H-NMR (392 MHz, CHLOROFORM-D) δ 4.61-4.68 (m, 2H), 4.12-4.31 (m, 4H), 1.64-1.71 (m, 2H), 1.24-1.41 (m, 9H), 0.87 (t, J = 6.7 Hz, 3H).

13C-NMR (99 MHz, CHLOROFORM-D) δ 167.72, 167.42, 166.68, 66.93, 66.91, 62.88, 62.81, 46.18 (d, J = 63.9 Hz), 46.15, 42.05 (d, J = 68.7 Hz),, 41.99, 31.67, 31.38, 28.34, 28.32, 25.45, 25.38, 22.74, 22.57, 14.21, 14.07, 13.93, 13.89.

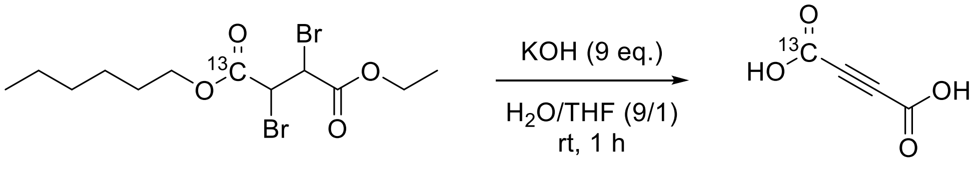

To a test tube containing KOH (2.52 g, 85% Wt, 9 Eq, 38.2 mmol) dissolved in water (3.8 mL) was added 1-ethyl 4-hexyl 2,3-dibromosuccinate-4-13C (1.65 g, 1 Eq, 4.24 mmol) dissolved in THF (0.4 mL) at 0 °C. The reaction was stirred vigorously at rt for 1 h. The reaction solution was washed with hexane and the aqueous phase was carefully acidified with 12N HCl (ca. 4 mL) at 0 °C. (*Caution: under strongly acidic conditions, hydrochlorination of the acetylene moiety takes place gradually*). The solution was washed with toluene to remove impurities. The organic materials were then extracted with AcOEt. The combined organic layers were dried over Na_2SO_4 and evaporated under vacuum to give but-2-ynedioic-1-^{13}C acid (328 mg, 2.85 mmol, 67.2 %, ~90% purity as determined by ^{13}C-NMR).

13C-NMR (100 MHz, DMSO-D6) δ 153.33, 75.50 (d, ^2J = 17.2 Hz), 75.45 (d, ^1J = 115 Hz).

HRMS (ESI) exact mass calculated for $C_3{}^{13}CH_2O_4$: m/z 113.9914 ([M-H]$^-$), found: m/z 113.9906 ([M-H]$^-$).

S-2. DFT simulations of Cp*Ru-catalyzed *trans*-hydrogenation in $CHCl_3$

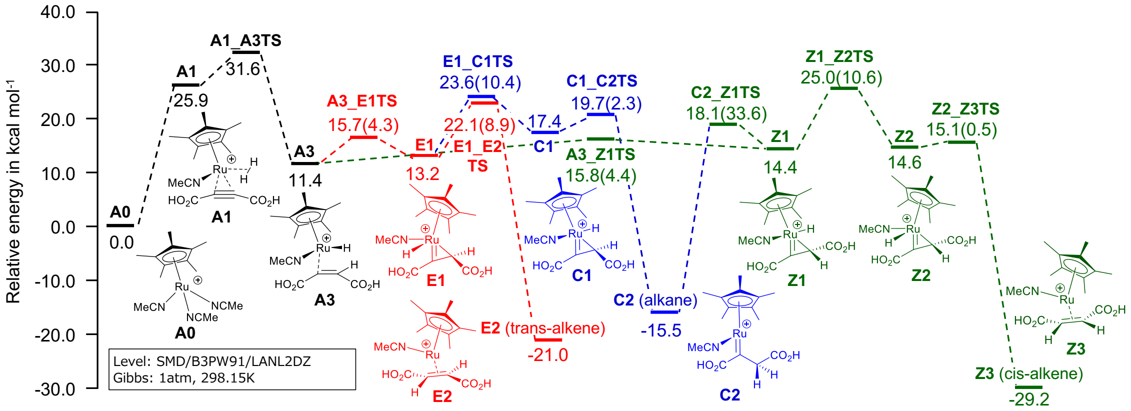

Free energy diagram for the hydrogenation of acetylene dicarboxylic acids using $[Cp^*Ru(MeCN)_3]PF_6$ in $CHCl_3$, instead of H_2O in main manuscript, was calculated at the SMD/B3PW91/LANL2DZ level of DFT theory. The free energy diagram was comparable to that of 2-butyne in CH_2Cl_2 using the same catalyst reported by M. Leutzsch et al. [1].

S-3. Further notes on the preparation of hyperpolarized [1-^{13}C] fumarate

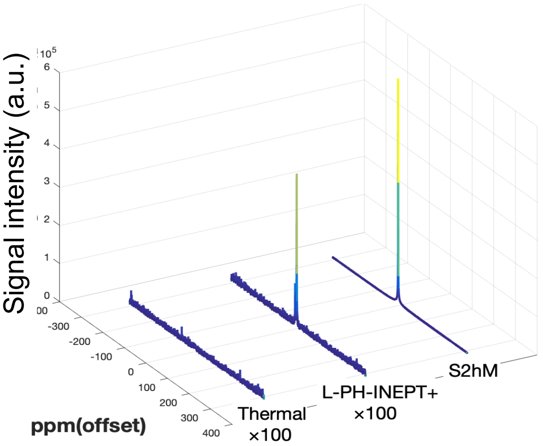

The achievable ^{13}C polarization of fumarate is dependent on the concentrations of both substrate and $[Cp^*Ru(CH_3CN)_3]PF_6$ catalyst. Higher concentrations of the catalyst (~ over 13 mM) result in higher concentration of the hydrogenated product fumarate but are accompanied by significant losses in ^{13}C polarization, perhaps due to accelerated longitudinal relaxation. Of note, the reaction rate of the hydrogenation of acetylenedicarboxylate using our experimental set-up is about 5—10 times slower than that reported by J. Eills et al [2, 3] even at the same concentrations of reactants, reaction temperature, pH, and parahydrogen gas pressure. As such, we needed to use longer reaction times to obtain high enough concentrations of fumarate for the *in vivo* studies reported in this work, which in-turn resulted in significant polarization loss. One noticeable difference from our study is that in Eills et al's study, a micro-sparger was used to make a micro-sized bubble of parahydrogen gas, which significantly increases the surface area of hydrogenation reaction and in turn the reaction rate.

In INEPT-type spin order transfer (SOT) experiments, the hyperpolarized ^{13}C spin must be

prepared longitudinally prior to *in vivo* studies to preserve the polarization. However, on our home-built 1.5T MR system, we observed unexplainable significant polarization losses when using 90-degree pulses at the end of the SOT sequence, perhaps due to imperfect B1 transmit performance. Performing the RF-induced polarization transfer within the imaging magnet – as per the SAMBANEDA-type approach – requires a complicated experimental set-up wherein both the SOT system and animal are simultaneously placed in the same MRI magnet, and more specifically, within the highly homogenous B_0 region. To circumvent this technical challenge, we opted to use MFC-based SOT approaches for *in vivo* experiments in this study.

S-4. Removal of $[Cp^*Ru(CH_3CN)_3]PF_6$ catalyst by QuadraPure® TU column

Almost all of the $[Cp^*Ru(CH_3CN)_3]PF_6$ catalyst can be removed by the column filter containing QuadraPure® TU if the hyperpolarized ^{13}C fumarate solution is passed very slowly (~ a minute) through the filter. However, in order to minimize polarization loss for *in vitro* and *in vivo* studies, rapid filtration is required, which was measured to result in ~0.1 mg/mL of the catalyst remaining in the final injection solution.

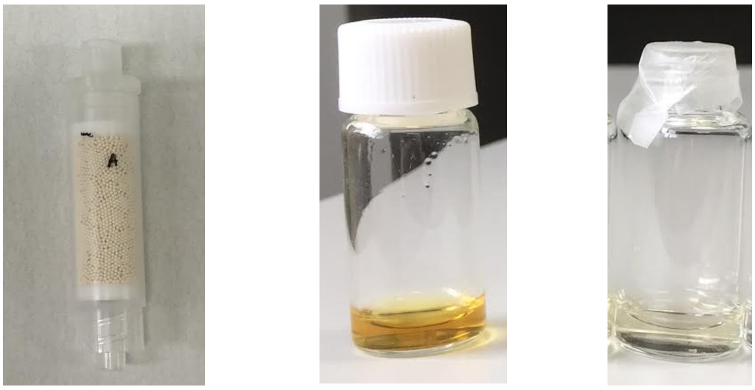

QuadraPure® TU inline column | before column | after column (rapid filtration)

References

[1] M. Leutzsch, L. M. Wolf, P. Gupta, M. Fuchs, W. Thiel, C. Fares, A. Furstner *Angew Chem Int Ed Engl.* **2015**, *54*, 12431-12436.

[2] J. Eills, E. Cavallari, C. Carrera, D. Budker, S. Aime, F. Reineri *J Am Chem Soc.* **2019**, *141*, 20209-20214.

[3] S. Knecht, J. W. Blanchard, D. Barskiy, E. Cavallari, L. Dagys, E. van Dyke, M. Tsukanov, B. Bliemel, K. Münnemann, S. Aime, F. Reineri, M. H. Levitt, G. Buntkowsky, A. Pines, P. Blümler, D. Budker, J. Eills *Chem RXiv. Preprint.* **2020**, DOI:10.26434/chemrxiv.12909989.v12909981.
